# With a biomechanical treatment in knee osteoarthritis, less knee pain did not correlate with synovitis reduction

**DOI:** 10.1186/s12891-017-1691-1

**Published:** 2017-08-10

**Authors:** Vikram Swaminathan, Matthew J Parkes, Michael J Callaghan, Terence W O’Neill, Richard Hodgson, Andrew D Gait, David T Felson

**Affiliations:** 1 Arthritis Research UK Centre for Epidemiology, Institute of Inflammation and Repair, Faculty of Medical and Human Sciences, Manchester Academic Health Science Centre, University of Manchester, Stopford Building, Oxford Road, Manchester, M13 9PT UK; 2 NIHR Manchester Musculoskeletal Biomedical Research Unit, Central Manchester NHS Foundation Trust, Manchester Academic Health Sciences Centre, Manchester, UK; 3 Faculty of Health, Psychology, and Social Care, Department of Health Professions, Manchester Metropolitan University, Manchester, UK; 4 Central Manchester University Hospitals NHS Foundation Trust, Manchester, UK; 5 Department of Rheumatology, Salford Royal NHS Foundation Trust, Salford, UK; 6 Centre of Imaging Sciences, Institute of Population Health, University of Manchester, Manchester, UK; 7 Clinical Epidemiology Unit, Boston University School of Medicine, Boston, MA USA

**Keywords:** Brace, Patellofemoral, Osteoarthritis, Synovitis, Dynamic contrast enhanced, Magnetic resonance imaging

## Abstract

**Background:**

Braces are used to treat pain in patellofemoral joint osteoarthritis (PFJOA). In a trial, we previously reported pain improvement after 6-weeks brace use. The pain reduction did not correlate with changes in Magnetic Resonance Imaging (MRI) assessed Bone Marrow Lesion volume or static synovial volume. Studies show that changes in the synovium on dynamic contrast enhanced (DCE) MRI are more closely associated with symptom change than static synovial volume changes. We hypothesised change in synovitis assessed using dynamic imaging could explain the reduction in pain.

**Method:**

One hundred twenty-six men and women aged 40–70 years with painful radiographically confirmed PFJOA were randomised to either brace wearing or no brace for 6-weeks. Pain assessment and DCE-MRI were performed at baseline and 6 weeks. DCE data was analysed using Tofts’s equation. Pain measures included a VAS of pain on nominated aggravating activity (VAS_NA_), and the KOOS pain subscale. Paired t-tests were used to determine within person change in outcome measures and Spearman’s correlation coefficients were used to determine the correlation between change in pain and change in the DCE parameters.

**Results:**

Mean age of subjects was 55.5 years (SD = 7.5) and 57% were female. There was clear pain improvement in the brace users compared to controls (VAS_NA_ − 16.87 mm, p = <0.001). There was no significant change to the dynamic synovitis parameters among brace users nor was pain change correlated with change in dynamic synovitis parameters.

**Conclusion:**

The reduction in knee pain following brace wearing in patients with PFJOA is not explained by changes in synovitis.

**Trial registration:**

Trial registration number UK. ISRCTN50380458/Registered 21.5.2010.

## Background

Patello-femoral Joint Osteoarthritis (PFJOA) is a common cause of knee pain in middle aged adults. In a recent randomised controlled trial of brace therapy in persons with symptomatic PFJOA we showed that a flexible sleeve knee brace resulted in a significant improvement in pain after 6 weeks and a reduction in bone marrow lesion (BML) volume in the PFJ [1]. The two structures in the knee that are both innervated by nociceptive fibers and reported to be causally related to knee pain are bone [2] and synovitis [3, 4]. In the bone, the most prominently assessed abnormality correlated with pain has been bone marrow lesions [5]. While hyaline cartilage pathology is the signature feature of osteoarthritis [6], this cartilage is not innervated, and it is not clear whether it is a source of pain. The change in pain during the trial was not correlated significantly with change in the BML volume, suggesting other mechanisms explain the pain reduction. Synovial tissue volume decrease has been linked with pain in observational studies [3, 4], but synovial volume assessed in the trial using static contrast enhanced magnetic resonance imaging (CE-MRI) did not shrink with the intervention [1].

Static measures of synovial volume may be less sensitive to pain than measures of synovial perfusion, assessed using dynamic contrast enhanced Magnetic Resonance Imaging (DCE-MRI) is a technique that utilises repeated imaging sequences of the joint whilst injecting contrast agent systemically [7]. This allows enhancement rates of tissues to be calculated as they are perfused by contrast agent. The rate of tissue enhancement has been shown to be more closely linked to active joint inflammation in rheumatoid arthritis (RA) than changes to static tissue volume measures alone, and correlates more strongly with change in pain following intra-articular steroid therapy [8]. We have recently shown that in knee OA, intraarticular steroid treatment leads to a major reduction in perfusion of the synovium as shown by DCE-MRI and that this reduction is far better correlated with pain reduction than measures of static synovial volume [9]. Since braces may diminish contact stress across the joint, leading to less microscopic damage and perhaps less need for the synovium to clear this debris, we hypothesised that the reduction in pain observed following brace use in our recent trial might be explained by changes in synovitis as assessed using DCE-MRI parameters. We therefore undertook a secondary analysis of the trial findings to examine this question.

## Methods

### Design

One hundred twenty-six subjects aged 40–70 years, recruited from primary and secondary care were randomly allocated to receive a patello-femoral sleeve brace immediately for 6 weeks or no brace. DCE-MRI was performed at both baseline (0 weeks) and at 6 weeks for all subjects. The number of subjects was chosen in advance to provide 80% power to detect an effect of the brace on knee pain (alpha = 0.05, two sided). Recruitment took place between August 2009 to September 2012 and the trial’s main results have been reported elsewhere [1]. The primary structural outcome of the brace trial was bone marrow lesion (BML) volume but pain improvement was not significantly correlated with change in BML volume. Our discovery after intraarticular steroid injection that dynamic changes in synovium during DCE-MRI correlated with pain change led us to ask the question as to whether these dynamic changes in synovial volume explained the pain change in the knee brace study as a secondary analysis of these trial data.

### Inclusion/exclusion criteria

Subjects were included if they had knee radiographs with a Kellgren and Lawrence (KL) score of grade 2 or 3 in the PFJ. This had to be greater than the KL grade for the tibiofemoral joint. Subjects had daily pain for the previous 3 months which was sufficiently severe to score 40 or above on a 0–100 mm for the visual analogue scale of pain on nominated aggravating activity (VAS_NA_), and have PF symptoms (such as pain with stairs). Other inclusion/exclusion criteria for eligibility have been reported [1]. As contrast enhanced scans were used, subjects with renal dysfunction or undergoing dialysis were excluded.

### Intervention

Subjects remained on their usual OA treatment. The intervention consisted of a Bioskin Patellar Tracking Q Brace (Ossur UK, Manchester, England). This is a Lycra flexible knee support with an optional strap that can be pulled over the patella; no difference in efficacy between the strapped and unstrapped configuration has been reported [10]. Simple randomisation was carried out by the study statistician with allocation using opaque envelopes. There is no known minimal required use to obtain the therapeutic effect of a knee brace, and we arbitrarily chose at least 4 h/day, so that if randomised to brace therapy, participants were instructed to wear it for a minimum of 4 h daily.

### Assessments

#### Knee pain

Following subject recruitment and randomisation, both groups completed questionnaires evaluating their PFJ pain and imaging with DCE-MRI. After 6 weeks, pain evaluation and DCE-MRI was repeated for all subjects in both groups. The primary symptom outcome measure was pain on a VAS_NA_ (0 mm = no pain, 100 mm = worst pain) and the Knee Osteoarthritis Outcome Score (KOOS) pain scale was a secondary outcome measure.

### Outcome measures: Structural

#### MRI assessment

DCE-MRI variables were collected for all subjects at baseline and after 6 weeks using the same magnet and scanning protocol. The enrolled knee only was scanned. Using a 1.5 T Philips Gyroscan ACS NT (Philips, Best Netherlands), axial proton density weighted (PDW) fat saturated (FS) repetition time approximately (TR) 5.5 ms, echo time approximately (TE) 1.9 ms, field of view (FOV) 14 cm × 14 cm, 256 × 256 matrix, slice thickness 3 mm sequences were obtained in all subjects, under the guidance of a musculoskeletal radiologist. The contrast agent Dotarem (gadoteric acid) was administered intravenously.

#### MRI analysis

The synovitis was segmented on the sagittal image, performed blinded, with computer image analysis excluding cartilage within the segmented area and calculating the proportion of synovial tissue within every voxel [11]. The segmentation was transferred onto the dynamic image using image registration techniques. Repeated MRI sequences were performed every 22 s, and contrast was injected following the 3rd sequence. In our analysis the primary outcome measure was change in enhancement rates of synovium between the baseline and 6-week visit. These variables act as a quantitative assessment of the change in severity of knee synovitis.

**Fig. 1:**
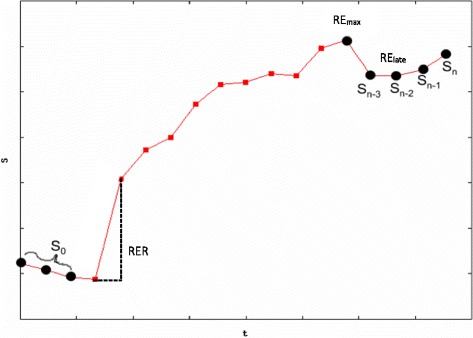
An example of a typical enhancement curve of a voxel of synovium during and post the administration of contrast agent. Three dynamic enhancement parameters are calculated from analysis of the enhancement curve; these variables (RER, RE_max_ and RE_late_) are labelled on the curve. The x-axis is time in seconds. There are 22 s between each plotted point on the enhancement curve. The y-axis shows signal intensity, starting at zero

Three DCE parameters used in this study were calculated from analysis of the synovial enhancement curve (EC) (Fig. 1) and one was calculated from the Standard Tofts’s equation [12]. The relative enhancement rate (RER) is a measurement the initial slope of the EC, which is sensitive to synovial vascularity and capillary permeability which reflects inflammatory activity. The maximum relative enhancement (RE_max_) and late relative enhancement (RE_late_) were also measured from the EC. K^trans^, the volume transfer coefficient calculated from Tofts’s equation, also depends on synovial vascularity and capillary permeability. It is thought as synovitis severity increases, DCE parameters will increase correspondingly [8, 12].

### Statistical analysis

Our analyses included a comparison of the brace and no brace groups from baseline to 6-weeks. Change in pain (VAS_NA_ and KOOS pain subscale) and the four DCE parameters were assessed by paired t-tests. Following this, unpaired t-tests were performed to identify differences in within person change between brace and control groups. Only subjects with complete data sets at each time point were included in analysis. In addition, bivariate Pearson’s correlation coefficients were performed to quantify the strength of associations between change in pain and change in the DCE outcomes in the brace group. The statistical analysis was performed using Stata (V.13.1; Stata Corporation, College Station, Texas, USA).

## Results

### Subjects

**Fig. 2:**
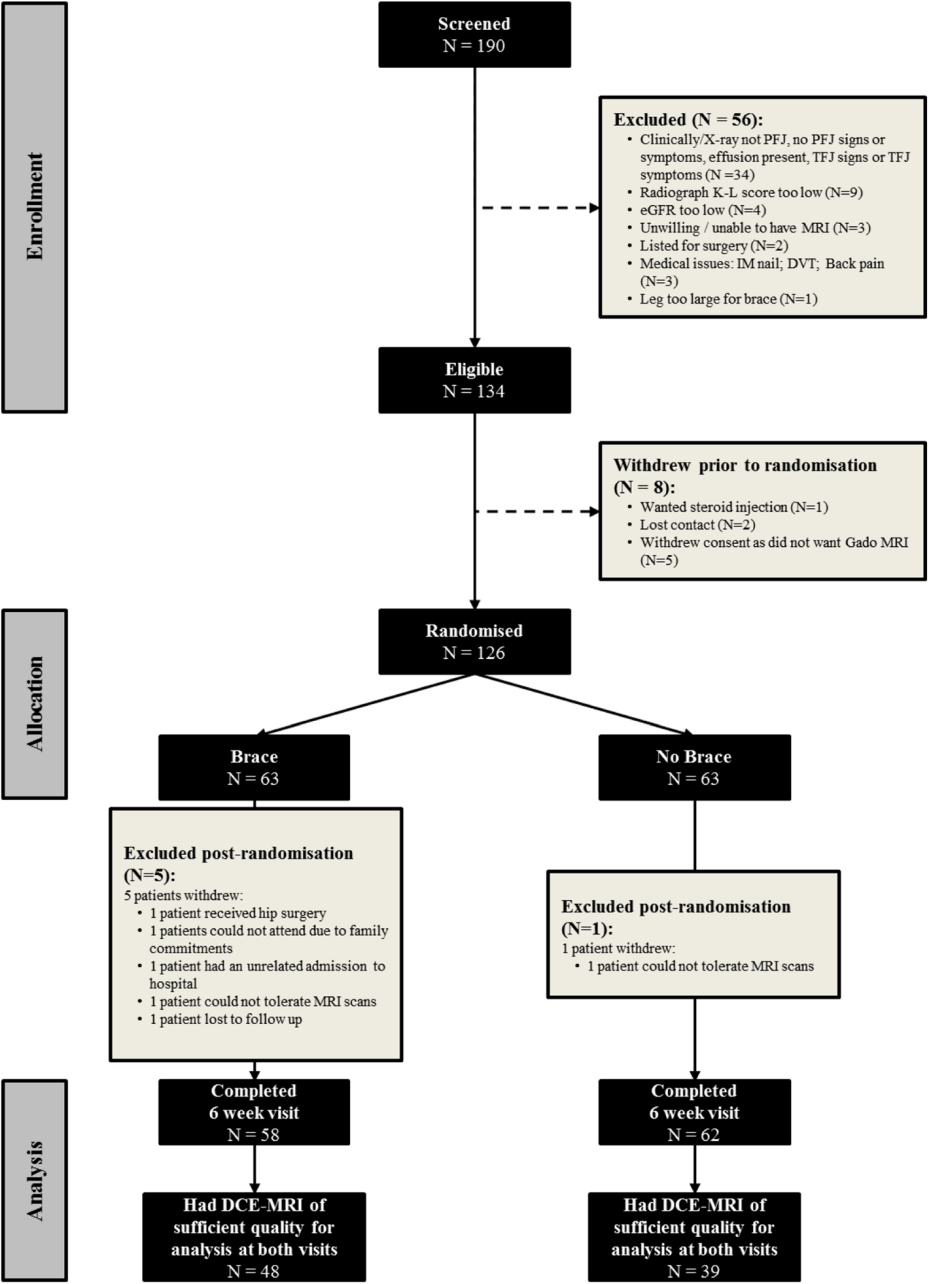
CONSORT Diagram for the BRACE trial’s 6 week period

One hundred twenty-six subjects were recruited; 63 were randomised to the intervention and 63 the control group (Fig. 2). The mean age of subjects in the intervention group was 54.5 years (SD = 6.7), body mass index (BMI) was 31.4 Kg/m^2^ (SD = 6.3) and 63.5% were female. The control group had a mean age of 56.4 years (SD = 8.1), BMI of 30.5 Kg/m^2^ (SD = 5.1), and 50.8% were female. Baseline pain was similar in both groups (mean VAS_NA_ 6.5). The brace group showed a mean pain reduction of 1.8 on the 10 cm VAS vs. 0 for the no brace group. The mean time the brace was worn was 7.35 h/day (SD = 3.10).

### Between group change in pain and synovitis

There was a significantly greater within person reduction in pain in the brace users compared to the controls over the 6-week period (VAS_NA_ *p* < 0.001, KOOS pain *p* = 0.01) (Table 1). In contrast there was no significant difference in within person change in most DCE-MRI parameters between the treated and control group. There was a significant between group difference in the within-group change in RER (RER change 0.008, *p* = 0.02), however, this was primarily due to the significant improvement in the control group (implying reduction in synovitis) rather than a positive change in RER seen in the intervention group (Table 2).

**Table 1:** Subject baseline characteristics - mean pain scores and DCE parameters of subjects in each group

	Controls (*N* = 63 randomised, 62 attended 6 week visit)			Brace group (*N* = 63 randomised, 58 attended 6 week visit)		
Variable	N in analysis	Baseline ean (SD)	6 week visit mean (SD)	N in analysis	Baseline mean (SD)	6 week visit mean (SD)
VAS_NA_	58^a^	6.17 (2.20)	6.30 (2.14)	56^a^	5.02 (2.55)	6.83 (2.13)
KOOS pain	61^a^	53.03 (18.27)	51.32 (18.30)	57^a^	57.72 (22.93)	48.94 (18.36)
K^trans^	48	0.016 (0.018)	0.019 (0.018)	39	0.018 (0.018)	0.017 (0.014)
RER	48	0.034 (0.015)	0.039 (0.020)	39	0.038 (0.018)	0.035 (0.010)
RE_late_	47^b^	2.084 (0.846)	2.186 (0.870)	39	2.128 (0.874)	2.118 (0.634)
RE_max_	48	2.474 (0.880)	2.635 (0.945)	39	2.569 (0.961)	2.523 (0.639)

^a^Number of observations for KOOS and VAS_NA_ are less than the number attending week 6 visit due to incomplete questionnaires

^b^Dynamic data model error prevented the use of one RE_late_ observation

**Table 2:** Subject baseline characteristics, mean within-group change following intervention with brace and in controls (without brace), and between-group differences in change (pre- and post-intervention)

	Controls (*N* = 63 randomised, 62 attended 6 week visit)			Brace group (*N* = 63 randomised, 58 attended 6 week visit)			Brace group - controls	
Variable	N in analysis	Mean change within group, (95% CI)	*P*-value	N in analysis	Mean change within group, (95% CI)	*P*-value	Mean change within group, (95% CI)	*P*-value
VAS_NA_	58^a^	−0.13 (−0.64 to 0.38)	0.61	56^a^	−1.82 (−2.39 to −1.24)	<0.001	1.69 (0.93 to 2.44)	<0.001
KOOS pain	61^a^	1.71 (−1.66 to 5.08)	0.31	57^a^	8.78 (4.36 to 13.20)	<0.001	−7.06 (−12.52 to −1.61)	0.01
K^trans^	48	−0.003 (−0.006 to 0.000)	0.05	39	0.001 (−0.003 to 0.005)	0.63	−0.004 (−0.009 to 0.001)	0.11
RER	48	−0.005 (−0.010 to 0.000)	0.04	39	0.003 (−0.002 to 0.008)	0.19	−0.008 (−0.014 to −0.001)	0.02
RE_late_	47^b^	−0.102 (−0.280 to 0.076)	0.25	39	0.010 (−0.175 to 0.195)	0.91	−0.112 (−0.367 to 0.142)	0.38
RE_max_	48	−0.161 (−0.357 to 0.036)	0.11	39	0.045 (−0.166 to 0.256)	0.67	−0.206 (−0.491 to 0.079)	0.15

^a^Number of observations for KOOS and VAS_NA_ are less than the number attending week 6 visit due to incomplete questionnaires. ^b^Dynamic data model error prevented the use of one RE_late_ observation

The reduction in RER represents a reduction in the rate of enhancement and was seen significantly more often in the control group, not in the treatment group

### Within group change in pain and DCE parameters

A significant reduction in the VAS_NA_ score (−18.16, *p* < 0.001) and an increase in the KOOS (8.78, *p* < 0.001) after 6 weeks brace use, signifying a reduction in pain. However, there were no significant changes to the DCE parameters in this group (Table 2). There was no statistically significant change to pain experienced over 6 weeks in the control group (VAS_NA_ *p* = 0.61, KOOS pain *p* = 0.31). There were small but significant changes noted to two of the DCE parameters in the control group; the mean change of K^trans^ was −0.003 (*p* = 0.05) and RER was −0.005 (*p* = 0.04), a reduction in enhancement rates of the synovium, suggesting a reduction in synovitis (Table 2).

Pearson’s correlations showed weak correlations between change in the dynamic parameters and the two pain measures (*r* = −0.10 to 0.12).

## Discussion

Our result suggest that synovitis, assessed using a sensitive imaging technique, does not improve following sleeve brace wearing. Change in synovitis does not explain the observed improvement in pain. Within the control group there was a small reduction in RER (*p* = 0.04) and K^trans^ (*p* = 0.05) variables over the study period suggesting a reduction in synovitis in this group, however, the magnitude of the changes was small and probably not clinically significant.

The DCE technique is a more sensitive method of detecting synovitis than static contrast enhanced imaging. Axelsen et al. demonstrated in 17 RA patients that intra-operative knee synovial biopsies showed histological inflammation which was highly correlated with changes in rates of synovial enhancement on pre-surgical T1 weighted MR images, especially the RER (spearman’s correlation coefficient = 0.70, *p* = 0.001) [8]. A review by Hodgson et al. was consistent with this; the RER was shown in multiple RA studies to correlate with histological, physiological and clinical disease activity changes [13]. Synovitis assessed using DCE-MRI was more strongly associated with change in pain following steroid injection than static MRI imaging [14].

As noted earlier, the two structures consistently linked to pain in knee OA have been bone marrow lesions and synovitis (bone attrition has also been linked to pain but would be unlikely to change in 6 weeks and its change is not readily measurable). The absence of any association with change in synovitis assessed using DCE-MRI suggests that the reduction in pain following brace wearing is not due to change in synovitis and that other mechanisms may be operating. What then is the mechanism for pain reduction? We did find in the trial that the patellar brace caused a reduction in BML volume in the patellofemoral compartment but that reduction was not significantly correlated with pain reduction. Although change in BML volume did not explain the pain reduction, it is possible that change in other structural features may have contributed. Ultimately, while pain improved, this was not necessarily accompanied by structural changes (although we may not have had the power to show that the reduction in BML volume was correlated with the pain reduction). The decrease in focal stress across the patellofemoral joint may have decreased nociceptive stimuli without causing a change in imaging parameters, and it is possible that our imaging approaches to pain are still too insensitive to detect the change induced.

This study was a secondary analysis of a trial of 126 patients. The original design did not include a formal sample size calculation for the DCE-MRI analysis. Instead, we made use of all available DCE-MRI scans collected during the course of the trial. Given the observed DCE-MRI trial data, we can calculate an estimated sample size for a future trial of the same design. Using RER as the primary outcome, the observed RER values for the change in the brace (mean change = +0.003; SD = 0.014) and no-brace group (mean change = −0.005; SD = 0.016), alpha of 0.05 and 80% power, a parallel-groups clinical trial testing for differences in the change in RER between the brace and no-brace group after 6 weeks would require 59 patients per group, a similar number to the trial findings presented here. A design using K^trans^ as the primary outcome, using observed values for the brace and no-brace group, would require 137 patients per group. Based on these estimates, it is possible but unlikely that our study was null because of inadequate power. We note that we found significant effects of dynamic measures of synovitis but in the opposite direction expected and that pain reduction effects were highly statistically significant.

The reduction in pain seen in the trial was modest but exceeded the minimum clinically important difference reported by Angst et al. (1.3 on a 10 cm scale) [15].

There are some limitations to be considered. It is possible that the six weeks trial duration may not have been long enough to identify significant physiological changes to synovium in participants who used a brace. This seems unlikely as changes are seen within 2-weeks following an intra-articular steroid injection [11, 16]. DCE-MRI was taken from a fixed region of interest (ROI) in the knee set on computer software. This is due to the standard Tofts’s equation requirements and the need to select a fixed area of tissue to make each image comparable between subjects. As each participant did not have the same size knee, the ROI varied between participants. This may have led to the fixed ROI window not capturing all the synovitis in each knee. This discrepancy was not recorded; there may be a difference between the groups with the percentage of total synovitis measured. In addition, sampling was done at intervals longer than might be optimal to detect dynamic change. The low temporal resolution of the DCE-MRI sequence (22 s) limits the accuracy of the model, particularly for estimating K^trans^. The field of view (FOV) was also limited reflecting the compromise in DCE-MRI between temporal resolution, spatial resolution and FOV in the phase encode directions; these could be improved for measurement of RE_late_ where high temporal resolution is less important. Also, movement between dynamic images may degrade reproducibility of measurements such as RER. This could be reduced by use of image registration (4).

## Conclusion

Brace therapy in symptomatic knee PFJOA is linked in the short term with a reduction in knee pain. There was no corresponding reduction in the observed DCE-MRI parameters of knee synovitis.

## Abbreviations

BML: Bone marrow lesion
CE-MRI: Contrast enhanced magnetic resonance imaging
DCE-MRI: Dynamic contrast enhanced Magnetic Resonance Imaging
FOV: Field of view
KL: Kellgren and Lawrence
KOOS: Knee osteoarthritis outcome score
K^trans^: Volume transfer coefficient calculated from Tofts’s equation
MRI: Magnetic resonance imaging
PFJOA: Patellofemoral joint osteoarthritis
RA: Rheumatoid arthritis
RE_late_: Late relative enhancement
RE_max_: Maximum relative enhancement
ROI: Region of interest
VAS_NA_: Visual Analog Scale of pain on nominated aggravating activity
